# Importance of Research in the Training of Thoracic Surgeons

**DOI:** 10.21470/1678-9741-2019-0290

**Published:** 2019

**Authors:** Hartzell V. Schaff, Anita Nguyen

**Affiliations:** 1 Department of Cardiovascular Surgery, Mayo Clinic, Rochester, Minnesota, United States of America.

Research in thoracic surgery is very broad and may involve molecular biology, integrated physiology, device development, or outcomes research. All of these areas are important to our specialty, but when should research training be undertaken and what are the benefits to the resident? The optimal timing for a research experience should be relatively early, after making a commitment to a career in thoracic surgery. For some, this may be after medical school, prior to internship and residency, and for others, it would be during the first years of surgical training. Although it is possible to gain research experience as a fully qualified thoracic surgeon, the clinical and administrative commitments of practice often take precedence, and special dedication and discipline are necessary for a surgeon to acquire education in research at a later stage in his or her career.

The ideal duration for a research experience may vary for different individuals. We believe that at least two years of dedicated time is required to gain the knowledge and important tools necessary to master a specific area of investigation, and to accomplish significant milestones in research, *e.g*. presentations and publications. It is important also to consider further formal education during a dedicated research experience. This should be tailored towards the trainee’s interests and the opportunities available at the resident’s institution. At Mayo Clinic, many residents and fellows pursue a postdoctoral Certificate or Master’s degree in Clinical and Translational Sciences, but other possibilities include Master’s and PhD programs in Biomedical Sciences and Public Health. Much can be learned from working on focused projects with surgical mentors, but participating in educational programs with formal curricula greatly expands the resident’s knowledge base.

There are several benefits of a research experience; one might be something as simple and practical as career advancement. The Resident may choose to go into the laboratory to improve his or her opportunity to obtain a residency position, post-residency fellowship, or academic staff position.

Another benefit of research is the satisfaction of the discovery and the recognition obtained through presentations and publications. Most residents spend five to seven years of medical school and residency absorbing as much general information in medicine and science as possible. In contrast, during a research experience, the trainee usually focuses on one problem in depth and can become an expert in that area.

For some, the research experience will be a direct lead into a career in investigation as a surgeon/scientist. The research fellow may continue investigation during clinical training and subsequently secure extramural funding. This might be considered the ideal outcome of resident research, but it happens to only a minority of residents who undertake dedicated time for research.

There is another rarely considered benefit of research; often, techniques and concepts mastered while doing research can be applied later to problems that are wholly unrelated to the original project. For example, during a research fellowship year, one of the authors worked in the laboratory of Dr. Vincent L. Gott, investigating methods of myocardial protection in a modified Langendorf preparation^[1]^. In this model, left ventricular function was measured with an intraventricular balloon, and we observed an increase in end-diastolic pressure when the balloon was reinflated, following an hour of ischemic arrest^[2]^. Reintroducing the same volume after arrest resulted in elevation of the end-diastolic pressure; this occurred in proportion to the decrease in systolic function.

Further experiments focusing on ventricular compliance demonstrated that the increase in end-diastolic pressure of post-arrest hearts was due to a smaller unstressed volume, rather than a change in compliance of the muscle (Figure 1). It appeared that ventricular chamber size was smaller in hearts injured by ischemia/reperfusion, and refilling the ventricles to prearrest volumes resulted in increased pressure^[3]^. These laboratory experiments led to an understanding of the important relationship between ventricular volume and diastolic function.

**Fig. 1:**
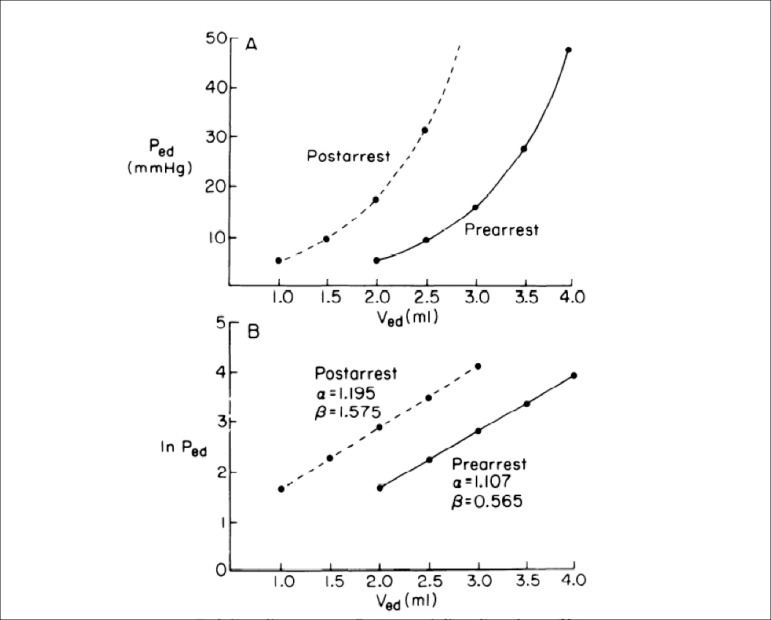
Experimental studies of ischemic arrest and reperfusion illustrate a mechanism of elevated end-diastolic pressure. End-diastolic pressure volume curves from a typical experiment in an isolated heart preparation demonstrate that prearrest and postarrest curves are exponential; postarrest diastolic pressure (Ped) is greater than prearrest diastolic pressure at any end-diastolic volume (Ved). As seen in the lower exponential plot, the end-diastolic pressure volume relationship is shifted upward and to the left and is not due to an increase in the slope or stiffness constant (α). Rather, the increased pressure is due to a reduction in the unstressed volume (β), i.e, a smaller cavity. Copyrighted and used with permission from the American Physiological Society^[7]^.

At Mayo Clinic, we evaluate and treat a large number of patients with hypertrophic cardiomyopathy (HCM). In one particular phenotype of HCM, patients have an apical distribution of left ventricular hypertrophy^[4]^. Management of patients with apical HCM who develop heart failure is difficult as medical therapy is rarely effective and cardiac transplantation is the only surgical option. We observed that the most severely limited patients with apical HCM had small ventricular chambers as a result of the apical muscle mass. Further, we speculated that the elevated end-diastolic pressure in patients might be the result of a small left ventricular chamber size, as was true for the isolated hearts that had been injured by ischemic arrest and reperfusion in the experimental laboratory.

It followed then, that surgical enlargement of the ventricle by apical myectomy might improve diastolic function in symptomatic patients with apical HCM and small left ventricular chamber size (Figure 2). In this procedure, the ventricle is enlarged through an apical ventriculotomy; muscle is removed from the septum, the anterior wall of the ventricle, and occasionally, the papillary muscles are shaved. First performed in September 1993, this operation has been employed in over 100 patients to relieve diastolic heart failure, and an informal comparison of these surgical patients with national HCM patients listed for heart transplantation demonstrated better survival in those undergoing apical myectomy (Figure 3)^[5-7]^.

**Fig. 2:**
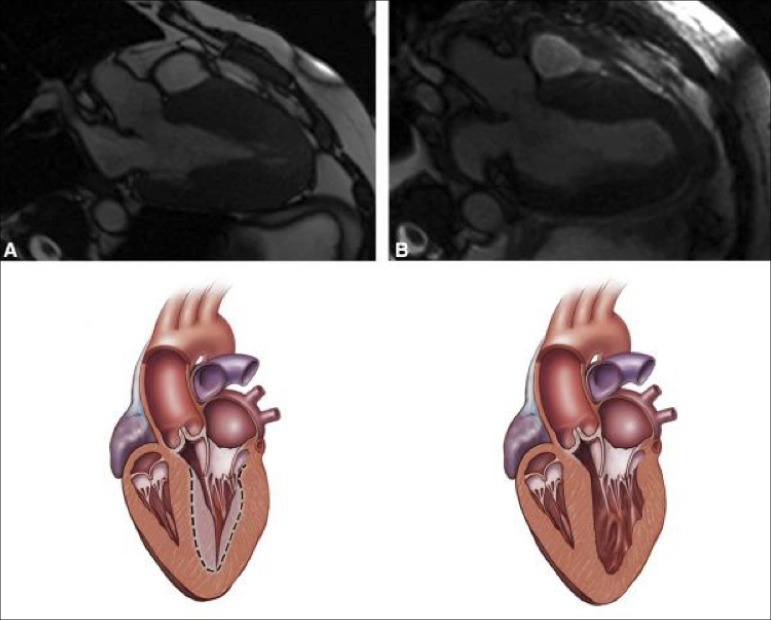
Panel A shows preoperative magnetic resonance image and illustration of a patient with apical hypertrophic cardiomyopathy. Panel B shows enlargement of the left ventricular cavity following apical myectomy. Copyrighted and used with permission from Elsevier Inc^[6]^.

**Fig. 3:**
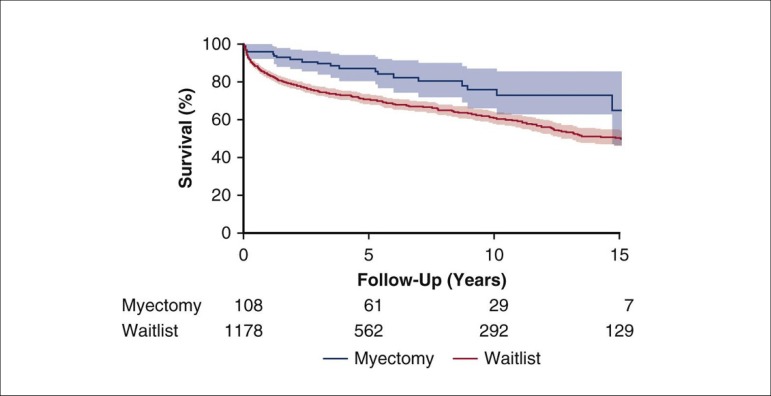
Survival of patients who underwent apical myectomy at Mayo Clinic (blue) and hypertrophic cardiomyopathy patients on a national transplant waitlist (red). Copyrighted and used with permission from Elsevier Inc^[6]^.

It is impossible to know whether this operation for apical hypertrophic cardiomyopathy would have been developed without the previous laboratory study of diastolic function. We suspect that the procedure would have been undertaken by someone at some time in the future, but recalling that the diastolic dysfunction in reperfused hearts may not be muscle stiffness per se, but reduced ventricular volume, led us to develop an operation for diastolic dysfunction in HCM. This was an unexpected benefit of a research, 20 years after an initial laboratory experience.

## References

[1] Schaff HV, Dombroff R, Flaherty JT, Bulkley BH, Hutchins GM, Goldman RA (1978). Effect of potassium cardioplegia on myocardial ischemia and post
arrest ventricular function. Circulation.

[2] Flaherty JT, Schaff HV, Goldman RA, Gott VL (1979). Metabolic and functional effects of progressive degrees of
hypothermia during global ischemia. Am J Physiol.

[3] Schaff HV, Goldman RA, Bulkley BH, Gott VL, Flaherty JT (1981). Hyperosmolar reperfusion following ischemic cardiac arrest:
critical importance of the timing of mannitol administration on preservation
of myocardial structure and function. Surgery.

[4] Binder J, Attenhofer Jost CH, Klarich KW, Connolly HM, Tajik AJ, Scott CG (2011). Apical hypertrophic cardiomyopathy: prevalence and correlates of
apical outpouching. J Am Soc Echocardiogr.

[5] Schaff HV, Brown ML, Dearani JA, Abel MD, Ommen SR, Sorajja P (2010). Apical myectomy: a new surgical technique for management of
severely symptomatic patients with apical hypertrophic
cardiomyopathy. J Thorac Cardiovasc Surg.

[6] Nguyen A, Schaff HV, Nishimura RA, Geske JB, Dearani JA, King KS (2019). Apical myectomy for patients with hypertrophic cardiomyopathy and
advanced heart failure. J Thorac Cardiovasc Surg.

[7] Schaff HV, Gott VL, Goldman RA, Frederiksen JW, Flaherty JT (1981). Mechanism of elevated left ventricular end-diastolic pressure
after ischemic arrest and reperfusion. Am J Physiol.

